# The mTOR Inhibitor Rapamycin Mitigates Perforant Pathway Neurodegeneration and Synapse Loss in a Mouse Model of Early-Stage Alzheimer-Type Tauopathy

**DOI:** 10.1371/journal.pone.0142340

**Published:** 2015-11-05

**Authors:** Robert Siman, Ryan Cocca, Yina Dong

**Affiliations:** Department of Neurosurgery, Center for Brain Injury and Repair, University of Pennsylvania Perelman School of Medicine, Philadelphia, Pennsylvania, USA; Nathan Kline Institute and New York University School of Medicine, UNITED STATES

## Abstract

The perforant pathway projection from layer II of the entorhinal cortex to the hippocampal dentate gyrus is especially important for long-term memory formation, and is preferentially vulnerable to developing a degenerative tauopathy early in Alzheimer’s disease (AD) that may spread over time trans-synaptically. Despite the importance of the perforant pathway to the clinical onset and progression of AD, a therapeutic has not been identified yet that protects it from tau-mediated toxicity. Here, we used an adeno-associated viral vector-based mouse model of early-stage AD-type tauopathy to investigate effects of the mTOR inhibitor and autophagy stimulator rapamycin on the tau-driven loss of perforant pathway neurons and synapses. Focal expression of human tau carrying a P301L mutation but not eGFP as a control in layer II of the lateral entorhinal cortex triggered rapid degeneration of these neurons, loss of lateral perforant pathway synapses in the dentate gyrus outer molecular layer, and activation of neuroinflammatory microglia and astroglia in the two locations. Chronic systemic rapamycin treatment partially inhibited phosphorylation of a mechanistic target of rapamycin substrate in brain and stimulated LC3 cleavage, a marker of autophagic flux. Compared with vehicle-treated controls, rapamycin protected against the tau-induced neuronal loss, synaptotoxicity, reactive microgliosis and astrogliosis, and activation of innate neuroimmunity. It did not alter human tau mRNA or total protein levels. Finally, rapamycin inhibited trans-synaptic transfer of human tau expression to the dentate granule neuron targets for the perforant pathway, likely by preventing the synaptic spread of the AAV vector in response to pathway degeneration. These results identify systemic rapamycin as a treatment that protects the entorhinal cortex and perforant pathway projection from tau-mediated neurodegeneration, axonal and synapse loss, and neuroinflammatory reactive gliosis. The findings support the potential for slowing the progression of AD by abrogating tau-mediated neurotoxicity at its earliest neuropathological stages.

## Introduction

There is considerable evidence that degeneration of the perforant pathway projection from entorhinal cortex to hippocampal dentate gyrus is an important contributor to the onset and progression of cognitive impairment in Alzheimer’s disease (AD). This pathway is a major source for excitatory innervation of hippocampus [1], a brain structure critical for memory [2,3]. Damage to the entorhinal cortex or perforant pathway projection in rats causes a rapid forgetting syndrome reminiscent of early-stage AD [4,5]. The perforant pathway is especially vulnerable in AD. The entorhinal layer II neurons of origin are among the first to develop aggregates of the microtubule-associated protein tau in the form of neurofibrillary tangles (Braak stage I; [6]) and the terminal field in the dentate gyrus molecular layer is a preferential early site for amyloid Aβ deposition [7]. Moreover, the pathway exhibits neurodegeneration and synapse loss in the elderly correlating with the transition from normal cognition to very mild cognitive impairment [8–11], and the neuronal loss progresses coincident with cognitive decline, until more than 90% of the pathway has degenerated [12,13]. The anatomical mapping of AD pathologies first supported the hypothesis that the disease process may extend along neocortical and subcortical interconnections [14]. Recent studies using spatially-restricted transgenic tau expression and microinjection of tau aggregates have provided evidence that a pathological form of tau itself may propagate through synaptically connected neural circuits, including via the perforant pathway [15–17]. Consequently, the identification and preclinical validation of therapeutic strategies for protecting the structure and function of the perforant pathway should provide significant new insights into the pathogenesis of AD, and is an important prerequisite for discovering drugs with a high likelihood of success as disease-modifying treatments for the early stages of AD.

AD is one of several neurodegenerative tauopathies for which tau oligomers and higher order aggregates have been linked to disease pathogenesis [18,19]. An important feature is tau hyperphosphorylation, which reduces its association with microtubules and promotes its propensity for aggregation. Whereas genetically modified mouse lines with cerebral amyloid deposition fail to recapitulate the extensive cortical neurodegeneration and synapse loss of AD (reviewed in [20]), a large body of evidence from cell-based and transgenic animal models of tau overexpression supports the neurotoxicity of hyperphosphorylated, aggregated tau [21–26]. A number of tau-based therapeutic approaches for AD are being explored directed at stabilizing microtubules, physically blocking tau aggregation, inhibiting protein kinases involved in tau hyperphosphorylation, and removing extracellular tau immunologically. Another promising strategy is to promote the degradation of tau multimers through stimulation of macroautophagy, a cellular pathway for clearance of protein aggregates and dysfunctional organelles. Stimulation of autophagy through either pharmacologic or molecular genetic inhibition of the protein kinase mechanistic target of rapamycin (mTOR) has been reported to reduce tau pathology [27–29], protect against neuronal loss [30], and lessen behavioral abnormalities [29,31] in tau-overexpressing transgenic mice. Unfortunately, a tau-directed therapeutic that preserves the structure and function of the perforant pathway has not been identified yet, a conspicuous omission given its importance to certain forms of long-term memory, preferential susceptibility in early-stage AD, and potential to spread tau pathology to limbic and neocortical regions.

Our laboratory developed a mouse model of early-stage AD-type tauopathy, based on an AAV vector that drives selective, robust, and sustained expression of mutant human tau or an eGFP gene as a control in the mouse lateral entorhinal cortex layer II and lateral perforant pathway projection. The model is characterized by tau hyperphosphorylation restricted to the perforant pathway neurons of origin in entorhinal layer II, along with tau-specific and gene dosage-dependent degeneration of the layer II neurons and deafferentation of the lateral perforant pathway terminal field, the dentate gyrus outer molecular layer [32]. Here, the model has been used to evaluate chronic systemic treatment with the mTOR inhibitor and autophagy stimulator rapamycin for its effects on tau-mediated degeneration, synapse loss, neuroinflammatory reactive gliosis, and activation of innate neuroimmunity in the mouse perforant pathway.

## Materials and Methods

### AAV-based gene delivery to the lateral entorhinal area and lateral perforant pathway

The identification of AAV vectors capable of driving focal, robust, and long-lasting expression of transgenes selectively in the mouse perforant pathway after unilateral microinjection into the lateral entorhinal cortex and methods for their generation have been described before, along with neurosurgical procedures for AAV delivery, pre- and post-operative animal care [32]. The vectors use the AAV2/9 hybrid serotype, the neuron-specific synapsin I gene promoter, and the WPRE post-transcriptional enhancer. They were produced, purified, sequence verified, and titrated by the University of Pennsylvania Viral Vector core facility in the Department of Genetics, under the direction of Dr. Julie Johnston. In the current study, 80 male mice of the CD-1 strain 3–4 months old (~35 grams) received stereotaxic microinjections of expression vectors for full-length human tau carrying 4 repeats and a P301L mutation (AAV-hTauP301L) or eGFP (AAV-eGFP) into the right lateral entorhinal area. All of the mouse procedures were reviewed and approved by the Institutional Animal Care and Use Committee of the University of Pennsylvania (Permit Number 801244), and are in full accord with both US Federal and EU Directive 2010/63/EU standards for animal experiments. Briefly, mice were deeply anesthetized with isoflurane. Sixty-two of the mice received 1.5 billion particles of the AAV-hTauP301L vector suspended in 0.5μl sterile saline by convection-enhanced delivery through a 27 gauge cannula fitted with a silicon tubing insert. Another group of mice was injected with 0.5 billion particles of AAV-hTauP301L (n = 6). Additional mice received 2 billion particles of AAV-hTauP301, 3 billion particles of AAV-eGFP, or 2 billion particles each of both viral expression vectors (n = 4/group). The mice were processed for biochemical analyses of protein and mRNA in brain homogenates at 3 weeks (n = 30) or for histological analyses at 3 weeks (n = 24) or 5 weeks (n = 26) after intra-entorhinal AAV vector delivery.

### Drug treatment

Rapamycin was purchased from LC Laboratories. A stock solution was prepared in ethanol and stored frozen at 50 mg/ml, then was diluted 20-fold immediately before use with 5% Tween-80/5% polyethylene glycol 400 in double deionized water. Ethanol was also added to the vehicle solution to 5%. Starting 4 days after delivery of 1.5 billion AAV-hTauP301L particles, mice received intraperitoneal injections of either ethanol-containing vehicle (n = 31) or rapamycin (n = 31) dissolved in vehicle at 15 mg/kg/day 3 times a week for either 3 or 5 weeks.

### Quantitative Western blot and qPCR analyses

Levels of pThr389-P70S6 kinase, a preferred phosphosubstrate of the mTOR kinase, as well as the native and cleaved forms of the autophagosomal protein microtubule-associated protein light chain 3 (LC3-I and–II, respectively) were measured in brain extracts, along with actin as a loading control. Hippocampal and neocortical extracts were prepared from the ipsilateral and contralateral regions from mice injected unilaterally with AAV-hTauP301L and treated for 3 weeks with either vehicle or rapamycin (n = 9/group). The regions were rapidly dissected and homogenized by probe sonication in ice cold Tris-HCl extraction buffer supplemented with protease and phosphatase inhibitors. Samples were diluted to 5 mg/ml total protein content, solubilized in electrophoresis sample buffer, heated to 90°C for 5 minutes, and fractionated on reducing, denaturing SDS polyacrylamide gels. Following blotting to nitrocellulose membranes, the proteins of interest were immunolabeled with near-infrared fluorophores and quantified by a LiCor imaging system, under conditions where band intensities varied in linear proportion with total protein load over a 3 order of magnitude range. The following primary antibodies were used: mouse anti-pThr389-P70S6 kinase (Cell Signaling Technologies; 1/1,000); rabbit anti-LC-3 (Abcam; 1/3,000); rabbit anti-actin (Abcam; 1/20,000); biotinylated mouse anti-human tau (Pierce; 1/3000). The species-specific fluorophore- and streptavidin-conjugated secondary antibodies were obtained from LiCor and diluted to 1/15,000.

For comparative analysis of human tau mRNA expression at 3 weeks after AAV-hTauP301L microinjection and systemic treatment with either vehicle or rapamycin (n = 6/group), ventral posterior blocks of hemi-forebrain either ipsilateral or contralateral to the vector injection were homogenized in TRIzol Reagent (Life Technologies) using a Potter-Elvehjem Teflon tissue homogenizer. RNA was extracted using TRIzol Reagent as described by the manufacturer. The aqueous phase from the TRIzol Reagent extraction was further purified using the RNA Clean and Concentrator kit as per the manufacturer protocol (Zymo Research). Total RNA (100 ng/μl) was converted to cDNA using the High Capacity cDNA Reverse Transcription Kit (Life Technologies) and amplified using exon spanning, highly specific TaqMan probes for human tau (MAPT) as the target gene (Hs00902190_m1) and mouse glyceraldehyde-3-phosphate dehydrogenase (Gapdh) as the housekeeping gene for normalization (Mm99999915_g1). Amplification was performed on the QuantStudio 12K Flex Real-Time PCR System (Life Technologies) and analyzed using the ΔΔC_T_ method. Specificity of the method for human but not mouse tau mRNA was confirmed by the >4 order of magnitude difference in signal between the hemispheres ipsilateral and contralateral to AAV-hTauP301L injection.

### Histological, histopathological, and morphometric methods

Brain tissues were prepared for histological analyses by transcardiac perfusion with freshly prepared ice cold 4% paraformaldehyde in phosphate buffer. To enable procedures for simultaneous immunohistochemistry and staining for synaptic zinc on the same brains, mice were initially perfused for 5 minutes with ice cold sodium sulfide solution [32]. Coronal 40 μm frozen sections were collected into 10 series, starting at the caudal entorhinal cortex and extending rostrally through the entire hippocampus. Immunohistochemistry was performed using a biotin-avidin indirect immunoperoxidase method previously described in detail [32–34]. The following antigens were labeled and antibodies used: total human tau (biotinylated mouse anti-human tau, phosphorylation state-independent clone HT7 at 1/1,000; Thermo); phospho-tau (biotinylated mouse anti-pTau202/205, clone AT8 at 1/1,000; Thermo); phospho-tau (biotinylated mouse anti-pTau231, clone PHF6 at 1/1,000; Thermo); NeuN (biotinylated mouse anti-NeuN at 1/3,000; Abcam), a neuronal nuclear marker; complement receptor 3/CD11b (rat anti-CR3 at 1/1,000; AbDSerotec), a microglial marker; glial fibrillary acidic protein (rabbit anti-GFAP at 1/5,000; Millipore), an astroglial marker; and immunoglobulin G (biotinylated goat anti-mouse IgG at 1/800; Vector), a marker for antigen-presenting microglia. The trans-synaptic transfer of human tau and eGFP expression to the dentate granule neuron targets of the perforant pathway was analyzed 5 weeks after AAV vector delivery. EGFP was visualized in the dentate gyrus under 1000x magnification with oil immersion, and z-stacks of fluorescence images captured and digitally sectioned by image deconvolution with NIS Elements software (Nikon). Seven mice were excluded from the histological analyses owing to either the absence of human tau expression (indicative of a clogged microinjection cannula) or AAV vector delivery that missed the lateral entorhinal area.

The integrity of lateral perforant pathway synapses was evaluated in one series of sections from each mouse using the Timm’s method to stain zinc, which is concentrated in presynaptic vesicles and differentiates the lateral perforant pathway synaptic field in the dentate outer molecular layer from neighboring synaptic zones in the middle molecular layer and stratum lacunosum moleculare of the hippocampal CA1 sector [35,36]. Some sections were stained with cresyl violet to visualize surviving neurons and non-neuronal cells in layer II of the lateral entorhinal cortex.

The survival of NeuN positive neurons of origin for the lateral perforant pathway in layer II of the lateral entorhinal cortex was determined by quantitative morphometry, using two different methods. In the first, a region of interest encompassing lateral entorhinal layer II and extending from the rhinal fissure ventrally to the border with the intermediate entorhinal area was identified by an experimenter blinded to the chronic drug treatment, immunopositive pixels defined by binary thresholding, and the percent area occupied by NeuN immunopositive neuronal nuclei compared between the regions contralateral and ipsilateral to the AAV vector microinjections [32]. The percentage of surviving lateral perforant pathway neurons was determined for each section as the ipsilateral/contralateral ratio times 100. At least 10 sections from 2 series covering the entire rostro-caudal extent of the entorhinal cortex were analyzed for each brain. For a second method, the number of NeuN immunopositive nuclei in layer II of the lateral entorhinal area was counted after binary image thresholding using NIS Elements software.

The same approach using a selected region of interest and binary thresholding was applied to morphometric analysis of microglial activation assessed by perforant pathway lesion-induced increases in complement receptor 3 (CR3) immunostaining in the dentate gyrus outer molecular layer. The tau-mediated increase in activated microglia was measured within the region of interest as the increase in the percent area occupied by CR3 positive pixels in the ipsilateral outer molecular layer compared to the contralateral region in each section.

The integrity of lateral perforant pathway synapses was determined from zinc staining of the dentate gyrus outer molecular layer as region of interest. The relative density of outer molecular layer presynaptic terminal zinc staining was compared between the contralateral and ipsilateral synaptic fields along the entire medial-lateral extent of both the suprapyramidal and infrapyramidal blades of dentate gyrus by an observer blinded to the drug treatment. The zinc staining intensity of the adjacent middle molecular layer was used to determine the background for subtraction. By comparing zinc staining intensity in relative units between the ipsilateral and contralateral outer molecular layer for every section, the influence of section-to-section and brain-to-brain variability in staining intensity was minimized.

The trans-synaptic transfer of human tau expression was quantified by counting HT7-immunopositive neurons in the ipsilateral dentate granule cell layer at 5 weeks after AAV-hTauP301L microinjection by an experimenter blinded to drug treatment. The total number of human tau immunopositive granule neurons in the 2 blades of the dentate gyrus was measured from at least 5 sections per mouse. Differences in each of the quantitative endpoints between vehicle- and rapamycin-treated mice were evaluated statistically by unpaired t-test.

## Results

### Chronic systemic rapamycin treatment reduces mTOR activity and stimulates a marker of autophagic flux in mouse brain

**Fig 1:**
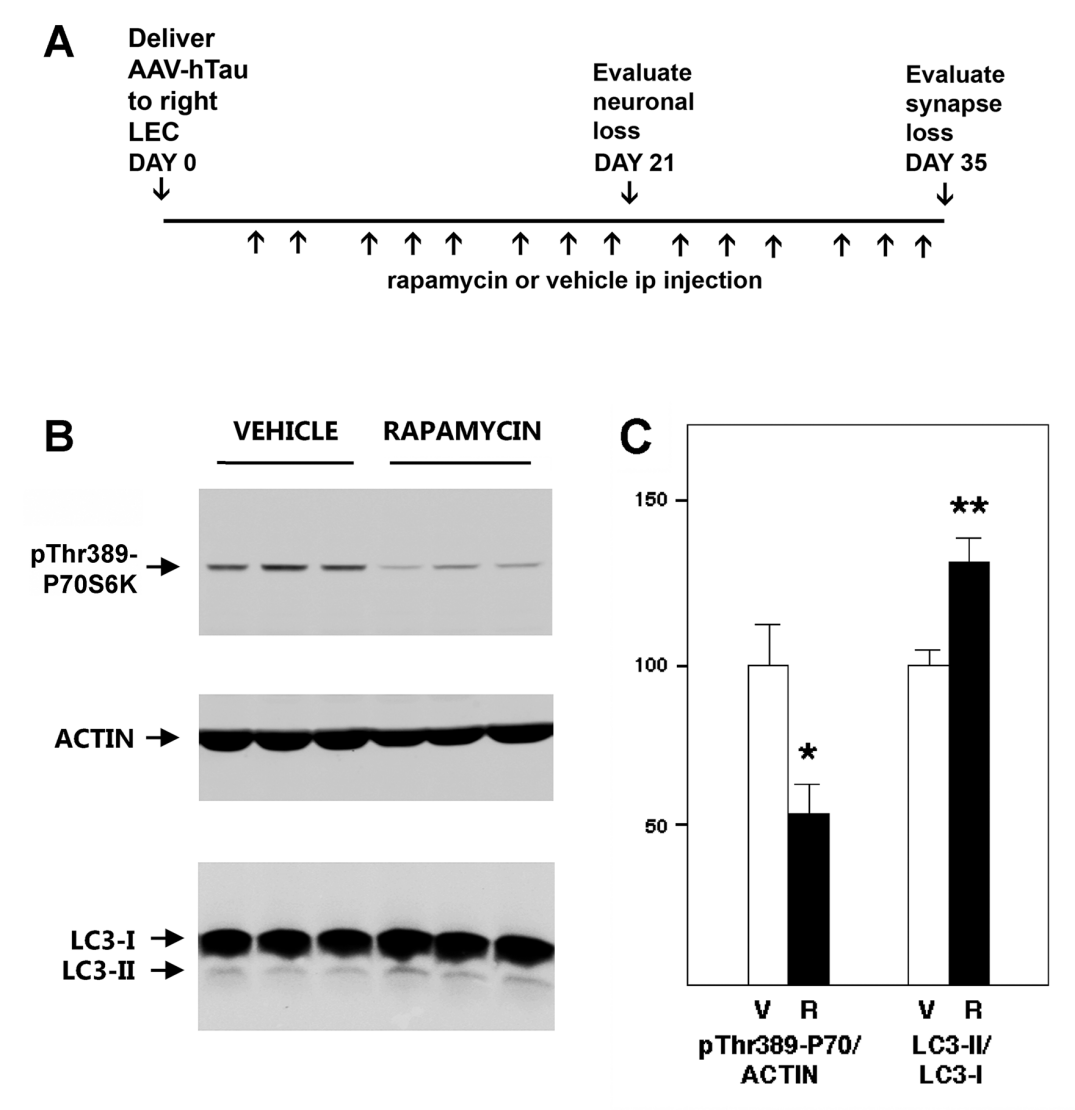
Chronogram for evaluating rapamycin in tau-mediated neurotoxicity for the perforant pathway, and biochemical evidence for target engagement in the mouse brain. Schematic of the time course for systemic treatment with rapamycin or vehicle after AAV-hTauP301L microinjections, in relation to the neuropathological assessments of the lateral entorhinal cortex (LEC) neurons and lateral perforant pathway synapses. (B) Western blot analysis of phosphorylation of the mTOR substrate P70S6 kinase on pThr389 and proteolytic generation of the autophagic vacuole membrane protein LC3 in hippocampus 3 weeks after viral vector delivery and drug treatment. Actin served as a loading control. (C) Quantitative analysis of P70S6 kinase phosphorylation and LC3 cleavage. Compared to hippocampus of vehicle-treated controls (V), chronic rapamycin (R) significantly attenuated mTOR substrate phosphorylation (*p = 0.013) and increased autophagic flux as monitored by cleaved LC3 (-II) relative to the full-length form (LC3-I; **p<0.01). Comparable results were obtained from the neocortex.

Rapamycin (also known as sirolimus) is an FDA-approved immunosuppressant drug that inhibits activity of the mTOR protein kinase in the rodent brain following peripheral administration [37], thereby triggering a signaling cascade that stimulates neuronal autophagy [38–41]. With an interest in evaluating the neuroprotective effect of rapamycin on the toxicity of pathological human tau for the mouse perforant pathway, we first analyzed markers of mTOR activity and autophagic flux in hippocampus after 3 weeks of rapamycin treatment (Fig 1A). Compared with vehicle, systemic rapamycin treatment partially blocked mTOR kinase activity in brain, as evidenced by a 48% reduction in phosphorylation of the mTOR substrate P70S6 kinase on Thr389 [42], in relation with actin as a loading control (p = 0.013; Fig 1B and 1C). To assess autophagic flux, we monitored processing of LC3 from its full-length (LC3-I) to its cleaved form (LC3-II), an important step in autophagosome membrane formation and autophagic flux [43]. Chronic rapamycin treatment increased hippocampal LC3-II levels relative to LC3-I in comparison with vehicle (p<0.01; Fig 1B and 1C). Comparable inhibition of P70S6 kinase phosphorylation and stimulation of LC3 cleavage were observed in the neocortex (data not shown). These marker changes provide confirmatory evidence that three weeks of peripheral rapamycin administration inhibits mTOR and stimulates autophagy in the mouse brain.

### Rapamycin protects entorhinal cortex from human tau-mediated neuronal loss

**Fig 2:**
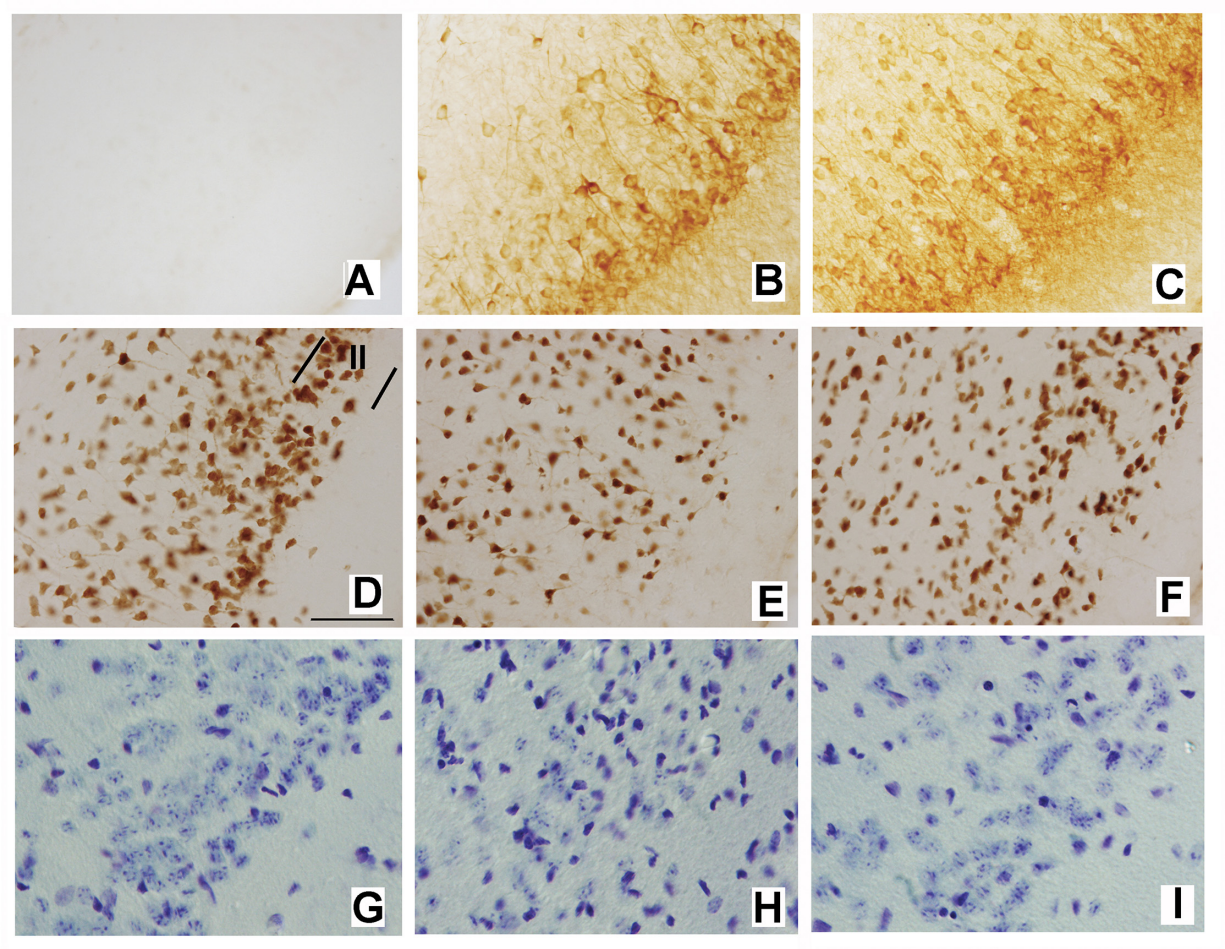
Protective effect of rapamycin for perforant pathway neurons of origin in layer II of the lateral entorhinal cortex. (A,D,G)–Lateral entorhinal cortex, contralateral hemisphere, vehicle treatment. (B,E,H)–Lateral entorhinal cortex ipsilateral to human tau gene delivery, vehicle treatment. (C,F,I)—Lateral entorhinal cortex, ipsilateral to human tau gene delivery, rapamycin treatment. (A-C) Immunohistochemical localization of pTau202/205 using the AT8 monoclonal antibody. Note that ipsilateral but not contralateral to the viral vector-based human tau gene delivery, pTau202/205 localizes preferentially in layer II neuronal perikarya and their proximal dendritic processes. Chronic rapamycin does not appreciably alter total pTau202/205 levels. (D-F) Immunohistochemical staining for NeuN-positive surviving lateral entorhinal neurons. Layer II of the lateral entorhinal cortex is denoted by the borders shown in D. Note the marked loss of layer II neurons after expression of pathological human tau (compare E with the contralateral hemisphere in D) and attenuation of the NeuN-positive neuronal loss by rapamycin treatment (F). (G-H) Analysis of neuronal survival and non-neuronal proliferation with cresyl violet staining. Compared to the control lateral entorhinal cortex (G), in entorhinal layer II ipsilateral to pathological human tau expression most of the healthy, plump, lightly-stained neuronal perikarya disappear, and are replaced by small, dark non-neuronal cells (H). Rapamycin preserves many of the healthy neurons and partially suppresses the non-neuronal cell proliferation (I). Scale bar = 75 μm.

Previously, we identified an AAV vector that after microinjection into the lateral entorhinal cortex drives localized, robust, long-lasting foreign protein expression preferentially in the layer II neurons as well as the perforant pathway projection terminating in the dentate gyrus outer molecular layer [32]. Using this vector, expression of hTauP301L but not eGFP as a control leads to rapid, gene dosage-dependent degeneration of perforant pathway neurons in layer II of the lateral entorhinal cortex, and loss of lateral perforant pathway afferents and synapses in the ipsilateral dentate gyrus. Here, we tested whether chronic systemic treatment with rapamycin could protect perforant pathway neurons from tau-mediated neurotoxicity. At 3 weeks after unilateral delivery of AAV-hTauP301L, tau phosphorylated on residues 202 and 205 (pTau202/205) was localized with the AT8 antibody, which marks an early stage of tau hyperphosphorylation. In lateral entorhinal cortex ipsilateral (Fig 2B and 2C) but not contralateral (Fig 2A) to gene delivery, pTau202/205 was localized predominantly in layer II neuronal perikarya and their proximal dendritic processes. Compared with vehicle treatment (Fig 2B), rapamycin did not appreciably and consistently alter phospho-tau labeling intensity or localization (Fig 2C); the same was true for total human tau labeled with a phosphorylation state-independent antibody specific for the human but not endogenous mouse protein, and for tau phosphorylated on Thr231 (data not shown). Immunostaining for the neuronal nuclear marker NeuN after treatment with vehicle revealed extensive tau-induced loss of neurons in layer II of the lateral entorhinal cortex in the ipsilateral hemisphere (Fig 2E) compared to the contralateral side (Fig 2D). Chronic rapamycin partially protected against the tau-mediated loss of NeuN-positive entorhinal layer II neurons (Fig 2F). The sparing effect of rapamycin for lateral perforant pathway neurons was confirmed by staining adjacent sections with cresyl violet. Compared with the contralateral entorhinal cortex (Fig 2G), human tau expression caused a near complete loss of the plump, healthy, lightly stained neuronal perikarya ipsilaterally in layer II, which instead were replaced by proliferation of small, dark non-neuronal cells (Fig 2H). Rapamycin partially protected the neuronal perikarya and markedly reduced the number of non-neuronal cells in layer II (Fig 2I). As shown by quantitative analysis, rapamycin decreased the tau-induced loss of area occupied by NeuN-positive lateral perforant pathway neuronal nuclei by 52% (Table 1; p<0.001), and the number of NeuN immunopositive neurons by 59% (data not shown; p<0.001).

**Table 1:** Systemic rapamycin attenuates pathological human tau-induced perforant pathway neuronal degeneration, synapse loss, trans-synaptic tau transfer, and reactive gliosis without influencing transgene expression.

Parameter	Vehicle	Rapamycin	% Effect	P Value
Entorhinal Neuronal	26.0 +/- 5.1	64.2 +/- 6.5	52	<0.001
Survival (% Contralateral)				
Perforant Pathway	61.7 +/- 14.4	5.2 +/- 3.1	92	0.007
Synapse Loss (Units)				
Granule Cell hTau	17.4 +/- 3.6	9.9 +/- 4.5	43	0.035
Transfer (neurons/section)				
Microglial CR3	284 +/- 48	139 +/- 12	79	0.009
(% Contralateral Dentate)				
Cortical hTau mRNA	.374 +/- .031	.310 +/- .049	17	0.38
(/GAPDH; Units)				
Cortical total hTau protein	2.03 +/- 0.09	1.83 +/- 0.14	10	0.47
(/Actin; Units)				

### Rapamycin inhibits the trans-synaptic spread of human tau expression to dentate gyrus, likely by reducing transfer of the AAV vector

**Fig 3:**
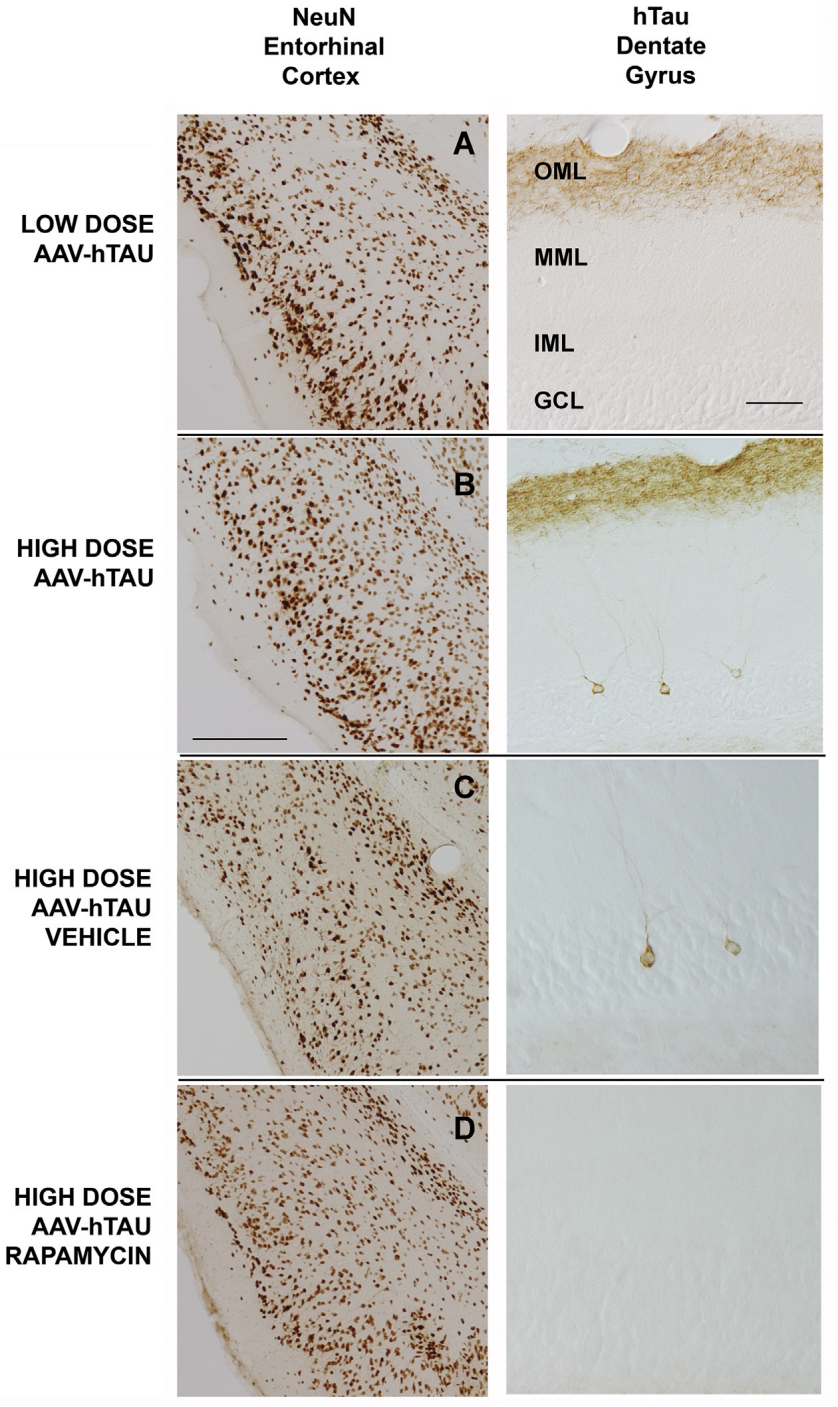
Dose-dependent tau neurotoxicity for lateral perforant pathway neurons is tied to trans-synaptic expansion of human tau expression, and both are reduced by rapamycin. Left column–NeuN immunohistochemistry in the lateral entorhinal cortex 5 weeks after AAV-based human tau gene delivery. Right column–Total human tau immunohistochemistry in the ipsilateral dentate gyrus. A) After delivery of 0.5 billion particles of AAV-hTauP301L (low dose), there is no appreciable neuronal loss in the lateral entorhinal layer II (see also Siman et al., 2013). In the dentate gyrus under these conditions, human tau is confined to the terminal field for the lateral perforant pathway in the outer molecular layer (OML). Additional abbreviations: MML- middle molecular layer; IML- inner molecular layer; GCL- granule cell layer. B) After delivery of 1.5 billion particles of AAV-hTauP301L (high dose), there is extensive loss of lateral entorhinal layer II neurons. In the dentate gyrus, human tau is present not only in the lateral perforant pathway terminal field in the OML, but also in scattered granule neurons in the GCL. C) Chronic treatment with vehicle does not appreciably alter the toxicity of pathological human tau for the layer II neurons of lateral entorhinal cortex or the spread of human tau expression to some of the dentate granule neurons. D) Chronic rapamycin treatment partially protects the perforant pathway layer II neurons from tau-mediated degeneration, and also partially inhibits the trans-synaptic spread of human tau expression to their granule neuron targets. Scale bar (left) = 200 μm; Scale bar (right) = 40 μm (A,B), 20 μm (C,D).

In our AAV model of early-stage AD-type tauopathy, human tau expression initially is confined to the neurons of origin in entorhinal layer II and the perforant pathway axonal projection as it traverses the hippocampal stratum lacunosum-moleculare (SLM), and terminates in the lateral perforant pathwayterminal field in the dentate outer molecular layer (OML). At later times, however, human tau expression spreads to the perforant pathway target neurons, the dentate granule cells, shortly after the pathway has degenerated [32]. Here, we investigated the effect of rapamycin on the trans-synaptic spread of human tau expression. At 5 weeks after delivery of a low dose of AAV-hTauP301L that is not acutely toxic to the perforant pathway (0.5 billion particles; [32]), NeuN staining revealed an intact layer II in the lateral entorhinal cortex (Fig 3A left). In dentate gyrus, human tau was confined to the perforant pathway terminal field in the OML (Fig 3A, right). In contrast, at 5 weeks after delivery of a higher, toxic dose of the tau vector (1.5 billion particles), lateral entorhinal layer II was depleted of surviving neurons (Fig 3B left), and in the dentate gyrus human tau expression expanded to include a small minority of granule neurons in the GCL (Fig 3B, right). Whereas chronic treatment with vehicle did not affect the layer II neuronal loss (Fig 3C left) or trans-synaptic spread of human tau expression (Fig 3C, right), rapamycin not only preserved the entorhinal layer II neurons (Fig 3D left) but also reduced the trans-synaptic expansion of human tau (Fig 3D, right). The number of human tau-expressing dentate granule neurons was reduced 43% by chronic rapamycin treatment (Table 1; p = 0.035).

**Fig 4:**
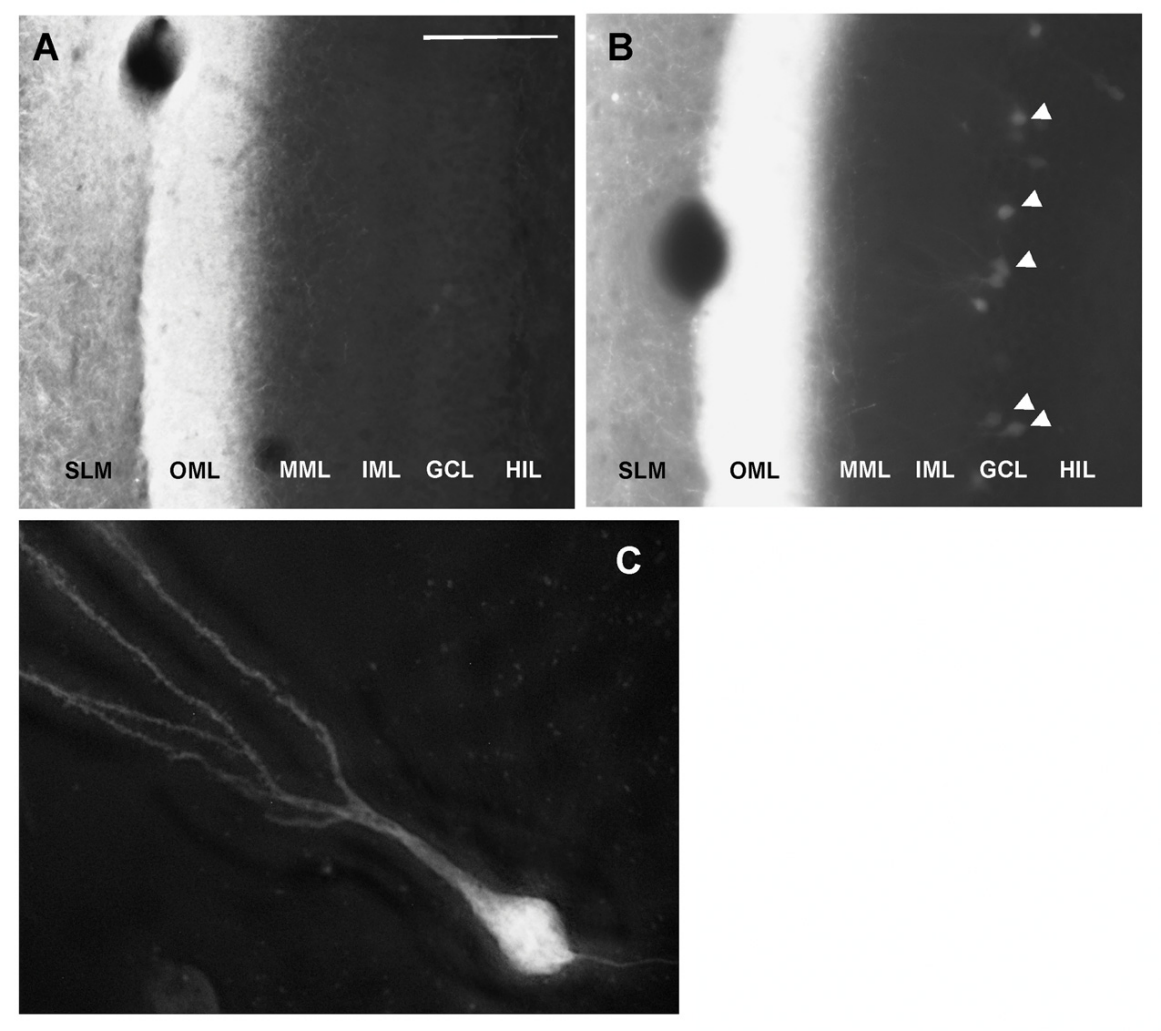
Co-delivery of AAV-eGFP with a toxic dose of AAV-hTauP301L to the lateral entorhinal cortex leads to trans-synaptic expression of eGFP as well as human tau in dentate granule neurons. A) After intra-entorhinal delivery of the eGFP vector alone, eGFP is expressed in the lateral perforant pathway projection as it traverses the stratum lacunosum-moleculare (SLM) of the hippocampal CA1 sector, before perforating the hippocampal fissure to form synaptic connections on dentate granule neurons in the outer molecular layer (OML). Note that the middle molecular layer (MML), inner molecular layer (IML), granule cell layer (GCL), and hilus (HIL) are devoid of eGFP. B) In contrast, when the eGFP vector is co-delivered to lateral entorhinal cortex along with a toxic dose of the pathological human tau vector, eGFP expression mimics human tau expression by expanding to scattered dentate granule neurons (arrowheads). C) Under high magnification image deconvolution microscopy, the eGFP-expressing cells in the granule cell layer are confirmed as dentate granule neurons based on their morphology. Scale bar = 150 μm (A,B), 15 μm (C).

There are two broad mechanisms by which human tau could spread trans-synaptically in a rapamycin-inhibitable manner. One possibility is that the human tau protein is transferred from the perforant pathway to its dentate granule neuron targets, a mechanism consistent with other evidence for trans-synaptic propagation of pathological tau through synaptically connected neural circuits [14–17]. A second possibility, however, is that the AAV vector rather than the human tau protein propagates trans-synaptically—a fraction of the AAV vector may be anterogradely transported to the dentate gyrus, and the vector released from the degenerating perforant pathway synapses to transduce dentate granule neurons. To distinguish between propagation of the human tau protein or the AAV vector in association with lateral perforant pathway degeneration, we co-injected entorhinal cortex with vectors encoding both human tau and eGFP. Delivery of 3 billion particles of AAV-eGFP alone was not toxic for the mouse perforant pathway, and eGFP was not transferred to dentate granule neurons for up to 5 weeks, despite its strong expression in perforant pathway axons in the hippocampal CA1 SLM and synapses in the dentate OML (Fig 4A; see also [32]). However, when 2 billion particles of AAV-eGFP were co-administered with a neurotoxic dose of AAV-hTauP301L particles, the localization of eGFP expanded at 5 weeks post-injection to include not only the perforant pathway axons in the SLM and synapses in the dentate OML, but also scattered dentate granule neurons (Fig 4B, arrowheads). The eGFP-positive cells in the dentate granule cell layer were confirmed as granule neurons based on their morphology (Fig 4C). These data suggest a mechanism for trans-synaptic spread of foreign protein expression in the AAV vector model whereby small amounts of the vectors undergo anterograde transport from the perforant pathway to dentate gyrus and are released upon tau-triggered degeneration, after which they transduce scattered dentate granule neurons to drive trans-synaptic protein expression. The dual effect of rapamycin to spare the perforant pathway from tau-mediated degeneration and lessen the trans-synaptic spread of human tau expression is consistent with this mechanism.

### Rapamycin protects lateral perforant pathway synapses from tau neurotoxicity

**Fig 5:**
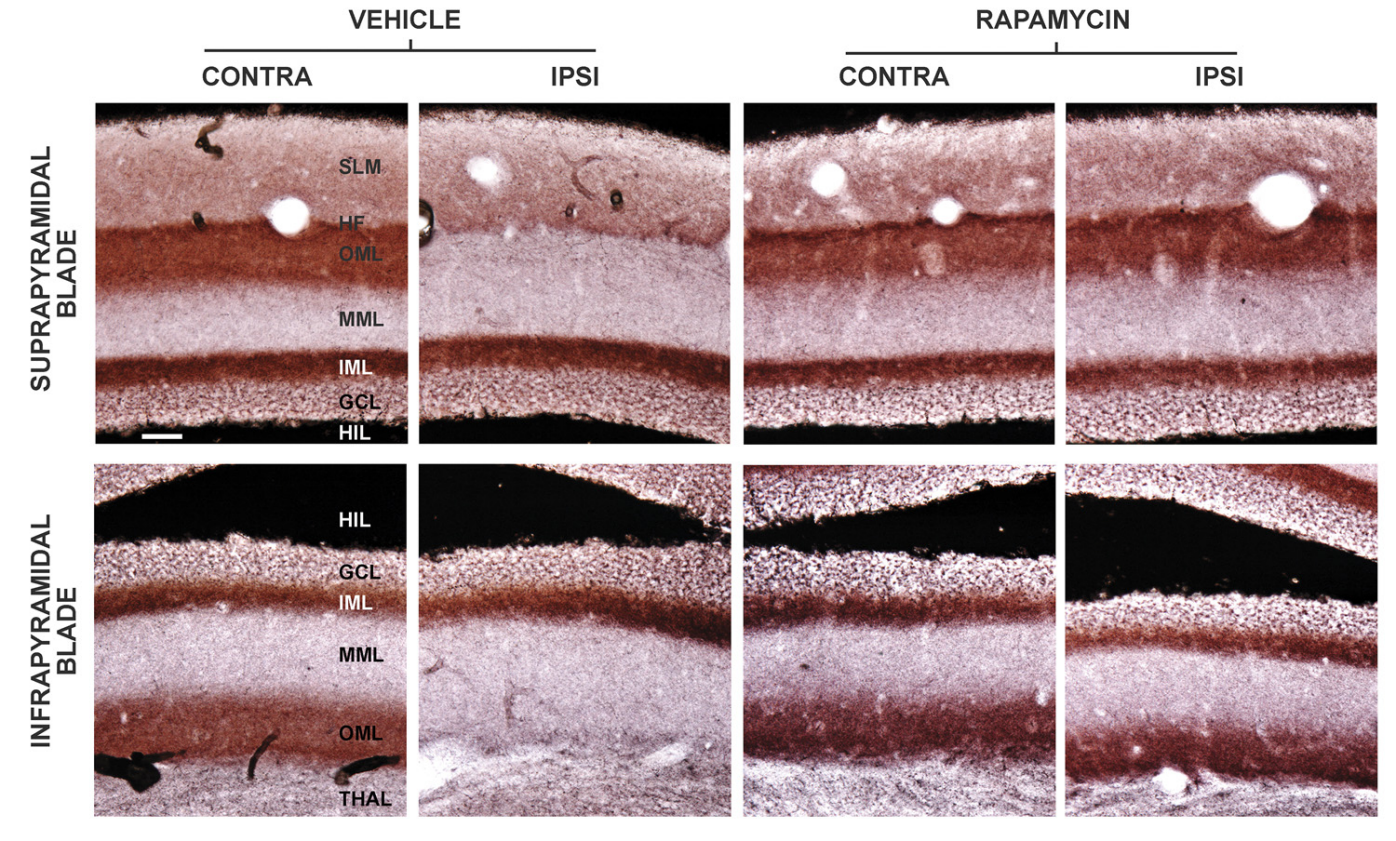
Rapamycin protects against tau-mediated lateral perforant pathway synapse loss. Timm staining in the dentate gyrus demarcates the afferent lamination of presynaptic terminal zinc. Left four panels, vehicle treatment: Contralateral to AAV vector injection (far left) in both blades of the dentate gyrus, zinc staining reveals a relatively dark zone in the outer molecular layer (OML) corresponding to the lateral entorhinal cortex and perforant pathway afferents. Neighboring afferent laminae in the stratum lacunosum-moleculare (SLM) above the hippocampal fissure (HF) for the suprapyramidal blade or the thalamus (THAL) for the infrapyramidal blade, or the middle molecular layer (MML) are demarcated by their lower synaptic zinc content. At 5 weeks after unilateral expression of pathological human tau in the lateral perforant pathway and treatment with vehicle, the lateral perforant pathway terminal field in the OML has largely degenerated in both blades of the ipsilateral dentate gyrus, concomitant with an expansion of the relatively zinc-poor medial perforant pathway afferents terminating in the MML. Right four panels, rapamycin treatment: Chronic rapamycin spares the relatively zinc-rich lateral perforant pathway synapses in the OML of both blades of the dentate gyrus. Scale bar = 40 μm.

To investigate further whether rapamycin protects the perforant pathway from human tau-induced synapse loss, we used the Timm’s method to stain hippocampus for zinc, which is concentrated within presynaptic vesicles and whose content distinguishes the relatively zinc-rich lateral perforant pathway presynaptic terminals in the dentate OML from the neighboring zinc-poor synaptic laminae in the dentate middle molecular layer (MML) and hippocampal SLM. As expected, for both the suprapyramidal and infrapyramidal blades, zinc staining in the contralateral dentate gyrus was much higher in the OML than in neighboring afferent laminae (Fig 5; [35,36]). At 5 weeks after delivery of AAV-hTauP301L in mice treated chronically with vehicle, the dense band of lateral perforant pathway synapses with high zinc content in the OML disappeared from both blades of the dentate gyrus, and the zone of faint synaptic zinc in the MML expanded, confirming our earlier report [32]. In sharp contrast, at 5 weeks after AAV-hTauP301L delivery coupled with chronic rapamycin treatment, the dense band of nerve terminal zinc persisted in the OML of both blades (Fig 5; compare the contralateral and ipsilateral OML). Based on quantitative morphometric analysis of synaptic zinc staining intensity in the OML, rapamycin reduced the tau-driven loss of lateral perforant pathway synaptic zinc by 92% (Table 1; p = 0.007).

A potential trivial explanation for the protective effect of rapamycin on tau toxicity for lateral perforant pathway neurons and synapses is that the drug could suppress expression of the human tau transgene. To investigate this possibility, human tau mRNA in mouse brain extracts was measured by quantitative real time PCR at 3 weeks after AAV-hTauP301L microinjection, and compared between vehicle- and rapamycin-treated mice. Chronic rapamycin did not significantly lower cortical human tau mRNA levels (Table 1). Similarly, rapamycin treatment did not significantly alter levels of total human tau protein, as measured by quantitative immunoblotting with the human-specific and phospho-independent monoclonal HT7 (Table 1). These data rule out suppression of tau transgene expression as the mechanism for its neuroprotective efficacy for the lateral perforant pathway.

### Rapamycin attenuates reactive gliosis induced by tau toxicity in the entohinal cortex and lateral perforant pathway terminal field

**Fig 6:**
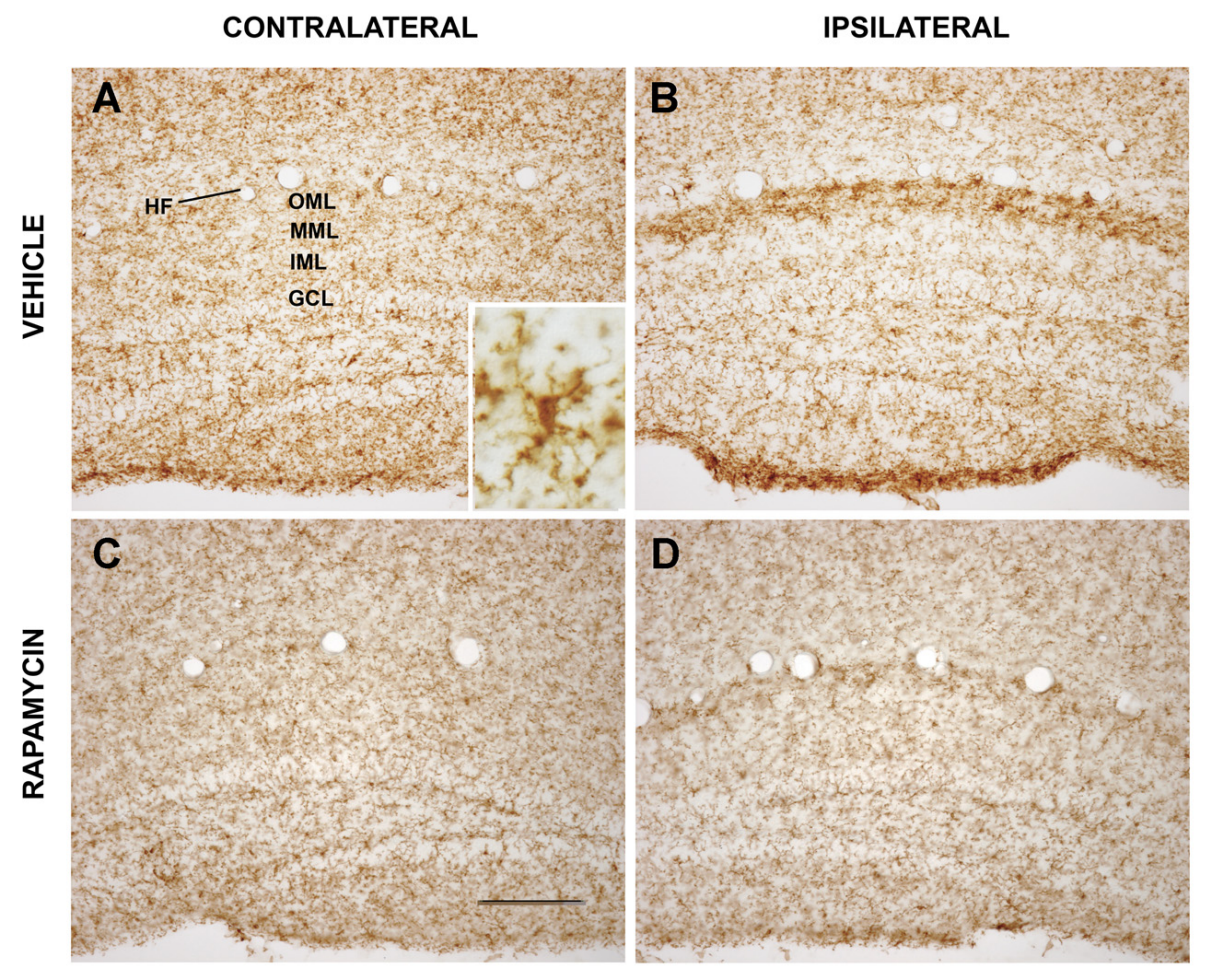
Rapamycin reduces pathological tau-triggered reactive microgliosis in the lateral perforant pathway synaptic field. Microglial activation triggered by pathological tau-induced neurodegeneration were distinguished from basal microglia based on their marked increase in immunohistochemical labeling for CR3/CD11b. (A) Cells with modest CR3 expression and the morphology of microglia (inset) were dispersed throughout the hippocampal dentate gyrus contralateral to viral vector delivery. (B) At 3 weeks after expression of pathological human tau in the lateral perforant pathway in mice treated with vehicle, dense bands of microglia with increased CR3 expression and enlarged processes were observed in the lateral perforant pathway terminal field in the outer molecular layer of both blades of the dentate gyrus. Whereas chronic rapamycin treatment did not appreciably alter basal CR3 expression in the contralateral hemisphere (C), it markedly attenuated the CR3 induction in reactive microglia in the lateral perforant pathway synaptic field (D). Abbreviations: HF- hippocampal fissure; OML- dentate outer molecular layer; MML- dentate middle molecular layer; IML- dentate inner molecular layer; GCL-dentate granule cell layer. Scale bar = 100 μm.

In response to brain damage, microglia and astroglia become hypertrophied, and increase expression of diverse neuroinflammatory, neuroimmune, and structural proteins. We examined the effects of tau-mediated perforant pathway toxicity and of neuroprotective rapamycin treatment on markers of reactive gliosis in the entorhinal cortex and lateral perforant pathway synaptic zone. Expression of complement receptor 3 (CR3; also known as CD11b) in the control hemisphere was widely distributed in cells with the morphology of microglial (Fig 6A and inset). Chronic rapamycin treatment did not appreciably alter microglial CR3 expression in the contralateral dentate gyrus (Fig 6C) or rest of the uninjected hemisphere (data not shown). On the other hand, at 3 weeks after AAV-hTauP301L gene delivery, a zone of enlarged microglia with markedly elevated CR3 expression developed in the lateral perforant pathway terminal field in the dentate OML (Fig 6B). Chronic rapamycin treatment markedly reduced the CR3 induction in this lateral perforant pathway synaptic zone (Fig 6D). In comparison with vehicle, rapamycin lowered the tau-mediated microglial induction of CR3 expression in the dentate OML by 79% (Table 1; p<0.01).

**Fig 7:**
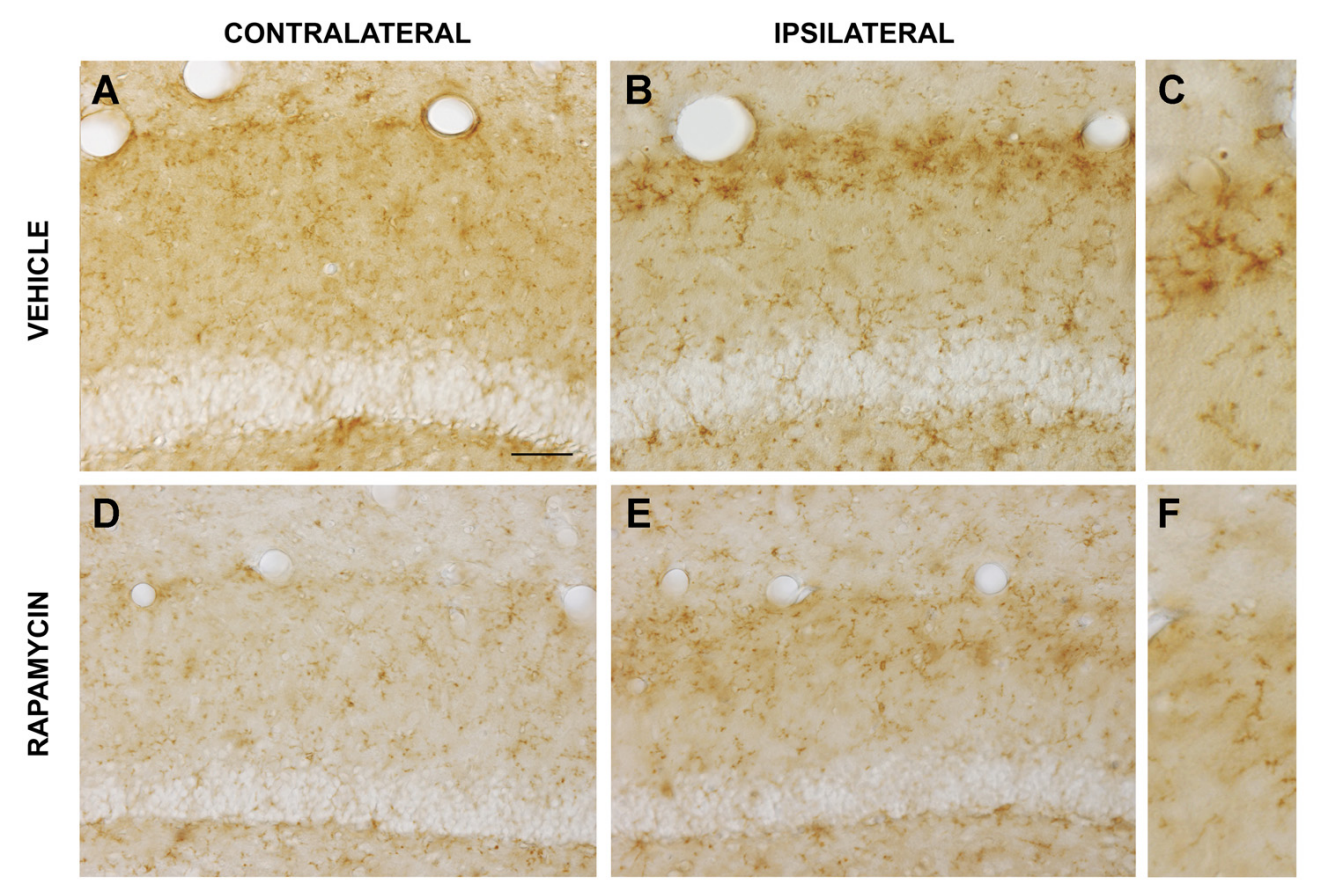
Rapamycin inhibits pathological tau-activated innate immunity in the lateral perforant pathway synaptic field. Microglial-mediated innate immunity in response to pathological tau-induced neurodegeneration was evaluated by immunohistochemical labeling for mouse IgG. (A) Cells with modest IgG expression were dispersed throughout the hippocampal dentate gyrus contralateral to viral vector delivery. (B) At 3 weeks after expressing pathological human tau in the lateral perforant pathway in mice treated with vehicle, a dense band of microglia with increased IgG expression was observed in the dentate gyrus outer molecular layer. (C) The morphology of IgG-expressing cells confirmed their identification as microglia. Whereas chronic rapamycin treatment modestly reduced microglial IgG expression in the contralateral hemisphere (D), it markedly attenuated the reactive microgliosis-associated increase in IgG expression in the lateral perforant pathway synaptic field (E, low magnification; F, high magnification). Essentially identical findings were made in the infrapyramidal blade of the dentate gyrus. Scale bar = 20 μm (A,B,D,E), 10 μm (C,F).

A subpopulation of microglia engages in antigen presentation and innate immune surveillance, and is identifiable by surface immunoglobulin G (IgG) expression [44]. We investigated the effect of tau-induced lesions of the lateral perforant pathway on microglial IgG expression and examined the influence of chronic rapamycin on tau-mediated activation of innate immunity. As shown in Fig 7A, IgG was expressed by scattered cells in dentate gyrus with short, thin, highly ramified processes characteristic of microglial morphology. Chronic rapamycin treatment lowered microglial IgG expression in the dentate gyrus (Fig 7D) and throughout the control hemisphere, consistent with its powerful immunosuppressant activity. At 3 weeks after expression of pathological human tau in the lateral perforant pathway, a band of microglia with increased IgG expression appeared in the denervated dentate OML (Fig 7B and 7C). Chronic rapamycin partially blocked the tau-induced increase in microglial IgG expression in the lateral perforant pathway terminal field (Fig 7E and 7F). Thus, rapamycin protected not only tau-mediated loss of lateral perforant pathway synapses as evidenced from presynaptic terminal zinc staining, but also inhibited the reactive microgliosis and activation of innate neuroimmunity that are triggered by the synaptic degeneration.

**Fig 8:**
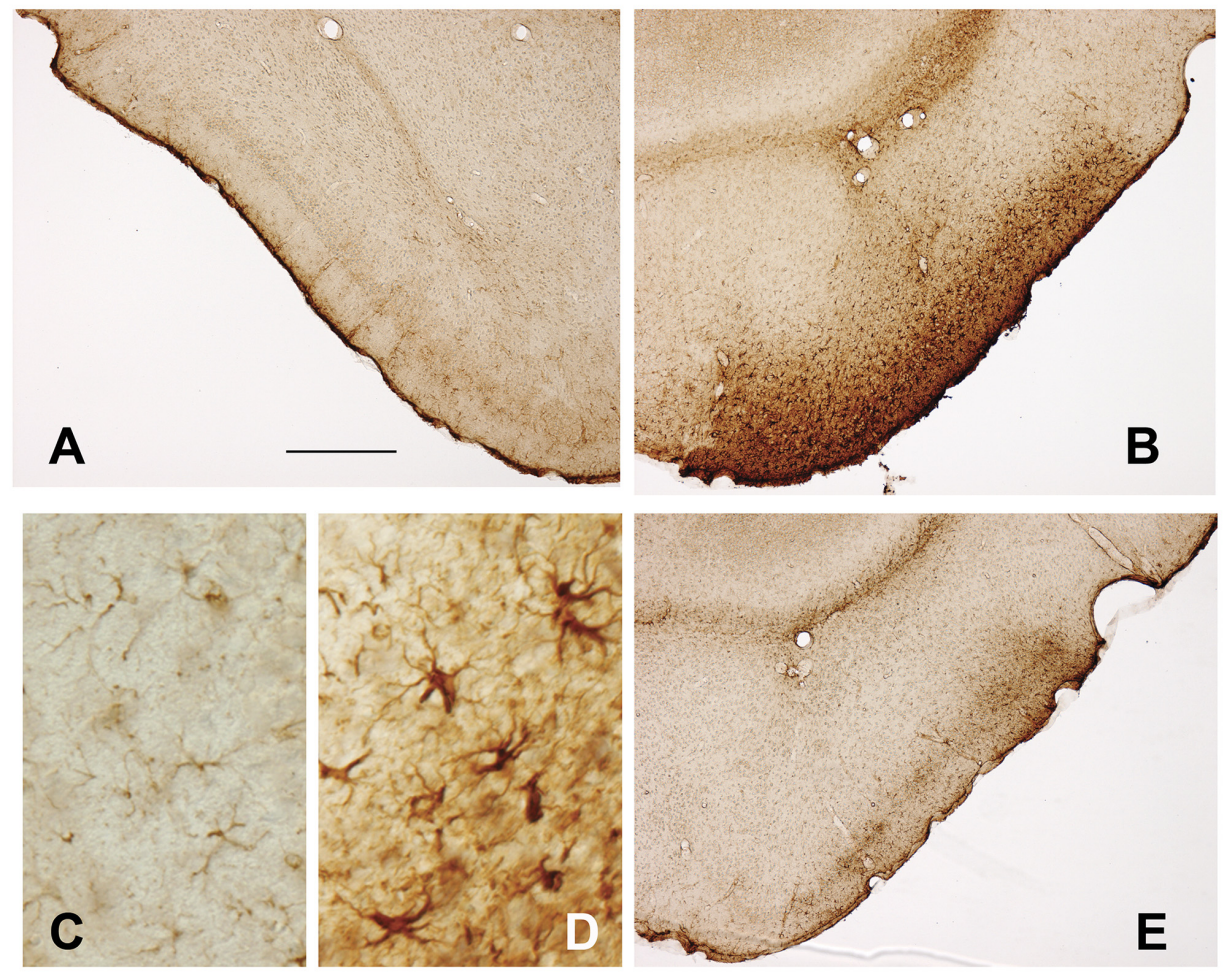
Rapamycin attenuates pathological tau-induced reactive astrogliosis in the lateral entorhinal cortex. Astroglial activation in response to pathological tau-induced neurodegeneration was evaluated by immunohistochemical staining for GFAP. (A,C) In lateral entorhinal cortex contralateral to viral vector-based gene delivery, astrocytes express relatively modest levels of GFAP (A, low magnification) and are small in size (C, high magnification). Ipsilateral to viral vector-based expression of pathological human tau and chronic vehicle treatment, the neuronal loss in layer II is accompanied by reactive astrogliosis, characterized by increased GFAP expression (B) and glial hypertrophy (D). In contrast, chronic rapamycin treatment markedly reduced the reactive astrogliosis in the pathological human tau-expressing lateral entorhinal cortex (compare E to B). Scale bar = 100 μm (A,B,E); 20 μm (C,D).

Reactive astrocytosis is also a prominent response to brain damage, and the phenotypic changes in astroglia contribute to injury-induced neuroinflammation [45]. To evaluate the effect of pathological human tau-driven neurotoxicity on the activation of pro-inflammatory astrocytes in the perforant pathway, immunostaining for glial fibrillary acidic protein (GFAP) identified reactive astroglia. At 3 weeks after unilateral AAV-hTauP301L delivery, the lateral entorhinal cortex contained abundant reactive astrocytes with strong GFAP expression in layer II and surrounding superficial layers (Fig 8B and 8D), compared with the uninjected, control entorhinal cortex (Fig 8A and 8C). In parallel with its protection against tau-mediated degeneration of layer II entorhinal neurons (Figs 2 and 3), rapamycin also markedly attenuated the reactive astrogliosis at the site of the lesion (Fig 8E).

## Discussion

Despite its recognized importance for long-term memory, susceptibility to developing tau pathology and degeneration very early in AD, and capacity for spreading tau-mediated neurodegeneration and pathology trans-synaptically, the perforant pathway has yet to be successfully targeted for neuroprotection against tau pathology. Here, we used a viral vector-based approach for inducing selective expression and toxicity of pathological human tau in the mouse lateral perforant pathway to evaluate the neuroprotective effects of rapamycin, an mTOR protein kinase inhibitor and autophagy stimulator. Expression of human tau bearing a pathogenic P301L mutation in layer II neurons of the lateral entorhinal cortex, but not eGFP as a control, causes rapid loss of these entorhinal neurons and their synapses in the dentate gyrus outer molecular layer within 3–5 weeks ([32]; Figs 2 and 3 and 5), and secondarily triggers reactive microgliosis and astrogliosis in the two regions (Figs 6–8). Based on a diverse set of qualitative and quantitative histological findings, chronic systemic rapamycin treatment significantly reduces all of these pathological effects of tau on the perforant pathway. The results raise the prospect of slowing the progression of AD pharmacologically by mitigating the toxicity of tau at one of its earliest neuropathological stages.

The protective effect of rapamycin for perforant pathway neurons originating in lateral entorhinal cortex layer II is demonstrated using multiple measures. Rapamycin partially inhibits the loss of lateral entorhinal layer II neurons expressing the neuronal nuclear marker NeuN, reduces the loss of healthy neuronal perikarya in layer II visualized by cresyl violet staining, and suppresses the lesion-induced proliferation of non-neuronal cells there (Figs 2 and 3). Additionally, the tau-driven degeneration of the lateral perforant pathway neurons is accompanied by activation of pro-inflammatory reactive astroglia in superficial lateral entorhinal cortex, an effect also attenuated by rapamycin (Fig 8). Collectively, the findings establish that chronic peripheral administration of rapamycin in the mouse protects a neuronal population from pathological tau-induced death that is especially susceptible to developing tauopathy and degeneration at the clinically symptomatic onset of AD.

We present strong evidence that rapamycin preserves not only the neurons of origin for the lateral perforant pathway from tau-mediated neurotoxicity, but also lateral perforant pathway axons and synapses innervating the hippocampal dentate gyrus. Four sets of experimental findings support the axonal and synaptic protection afforded by rapamycin. The first is based on staining for presynaptic vesicular zinc as a pathway-specific method for evaluating the integrity of the lateral perforant pathway projection and synapses from pathological human tau by systemic rapamycin treatment. This pathway-specific assessment is preferred over labeling for general synaptic markers, since perforant pathway lesions cause sprouting and synaptic replacement by neighboring afferents originating from other, unaffected brain regions [46]. Expression of pathological human tau almost completely eliminates the lamina of dense presynaptic zinc that delineates the lateral perforant pathway terminal field in dentate outer molecular layer, and rapamycin preserves this zone of synaptic zinc to near control levels (Fig 5). Secondly, pathological human tau triggers microglial activation in the synaptic field in the outer molecular layer as monitored by induced microglial expression of CR3/CD11b, and rapamycin attenuates the reactive microgliosis concomitant with its preservation of synaptic zinc (Fig 6). The microglial subtype that exhibits CR3 induction after brain damage expresses a number of pro-inflammatory cytokines and chemokines [47], so combined with its effects on reactive astrocytes in the entorhinal cortex, rapamycin attenuates activation of two cell types contributing to the neuroinflammation that follows neurodegeneration. While the contributions of neuroinflammatory reactive glia to progressive neurodegenerative diseases are not fully understood and are a topic of continuing debate [48–50], our data highlight the potential of rapamycin for protecting against not only the primary neurotoxicity of tauopathy, but also any secondary neuroinflammatory processes activated by tau toxicity that might contribute to bystander neuronal damage. Thirdly, tau toxicity for the lateral perforant pathway is accompanied by localized activation of innate neuroimmunity, manifested by increased microglial IgG expression in the dentate terminal field. Once again, rapamycin reduces this effect of pathological tau (Fig 7). Finally, the human tau expression spreads over time from the lateral perforant pathway to include the dentate granule neuron targets of the pathway, and rapamycin partially blocks this trans-synaptic expansion of human tau expression (Fig 3). Evidence from the co-injection of AAV vectors encoding for both hTauP301L and eGFP suggests that, in our model, the viral vectors themselves rather than the human tau protein spread trans-synaptically in response to perforant pathway degeneration (Fig 4). From its combined effects in the lateral perforant pathway terminal field to preserve synaptic zinc, attenuate reactive microgliosis, and inhibit trans-synaptic transfer of the AAV-hTau vector, rapamycin clearly protects against tau-mediated synaptotoxicity. In AD, loss of perforant pathway synapses occurs at the earliest clinically symptomatic stage, and progresses over time as a structural correlate for the magnitude of cognitive decline [11,13]. Therefore, the identification of rapamycin as a systemic treatment for protecting lateral perforant pathway neurons and synapses from tau-mediated degeneration has implications for therapeutic strategies attempting to slow the progression of AD at multiple stages of disease.

Stimulation of neuronal autophagy is a promising strategy for promoting the clearance of tau and other aggregation-prone proteins implicated in neurodegeneration. One reason we evaluated an mTOR inhibitor and autophagy stimulator as a protectant for the perforant pathway is the reported beneficial effects of stimulators of autophagy on neuropathology and behavior for other regions of the central nervous system in experimental models of tauopathy [27–31]. Autophagy stimulation can also mitigate pathology and improve behavioral function in mouse experimental models of diverse neurodegenerative disorders characterized by aberrant protein aggregation [51–54]. Although the functional status of neuronal autophagy is difficult to assess in the AD brain from post-mortem studies alone, the accumulation of autophagic vacuoles and autolysosomal hydrolases in vulnerable neuronal populations, along with reduced levels of at least some of the proteins required for autophagosome formation, suggest that defective autophagic flux may be tied to the protein deposition and neurodegeneration [55]. In the present study, the stimulation of autophagy by chronic systemic rapamycin, which is known to permeate the mouse brain after peripheral administration [37], is supported by its partial inhibition in the brain of P70S6 kinase phosphorylation, an established mTOR substrate, and increase in cleavage of LC3, a modification important for autophagosome formation. However, these data alone are insufficient to establish stimulation of autophagy as the predominant mechanism by which rapamycin protects the perforant pathway from tau neurotoxicity. As an inhibitor of the multiprotein complex mTORC1 and, to a lesser extent, mTORC2, rapamycin impacts signaling pathways that modulate, in addition to autophagy, cell survival, protein translation, lipid biosynthesis, mitochondrial proliferation, and energy metabolism [41]. A trivial effect of rapamycin on expression of the human tau transgene can be ruled out, based on its lack of effect on human tau mRNA or total protein levels (Table 1). Rapamycin is also a strong immunosuppressant, although its effects at ameliorating pathological tau-induced reactive gliosis and innate neuroimmunity are likely secondary to its primary effect of reducing degeneration of the lateral perforant pathway. Whereas other studies identified trehalose and methylene blue as additional autophagy stimulators capable of reducing the aggregation of hyperphosphorylated tau and behavioral abnormalities in tau transgenic mice, these drugs also have pleiotropic effects rendering their mechanisms of action difficult to ascertain definitively [29,30,56,57]. Finally, there is increasing evidence that the highly ordered neurofibrillary tangles of tau may not be responsible for its neurotoxicity [32,58,59]. The multimeric form of tau that is toxic for human brain neurons has yet to be defined, and so the influence of autophagy stimulators on the steady-state levels of tau variants that drive neurodegeneration is currently unknown. Further studies will be required using additional pharmacological and molecular genetic methods to define the autophagic and potentially other neuronal mechanisms by which rapamycin protects perforant pathway neurons and synapses from pathological tau.

In conclusion, this study identifies the mTOR inhibitor and autophagy stimulator rapamycin as a systemic treatment that mitigates pathological tau-driven loss of perforant pathway neurons, axons, and synapses, and reduces tau-induced neuroinflammatory processes. Given the fundamental importance of tauopathy and degeneration in the perforant pathway for the onset and clinical progression of AD, these data raise the possibility of slowing the progression of the disease pharmacologically at its earliest neuropathological stage. Careful delineation of the neuroprotective mechanisms of rapamycin in the perforant pathway, and additional insights into neuron-specific signaling mechanisms regulating the degradation of aggregation-prone pathological proteins should foster the development of novel therapeutic strategies for impeding the tau-mediated pathology and progressive brain atrophy in AD.
